# Prescription Stimulant Medical and Nonmedical Use Among US Secondary School Students, 2005 to 2020

**DOI:** 10.1001/jamanetworkopen.2023.8707

**Published:** 2023-04-18

**Authors:** Sean Esteban McCabe, John E. Schulenberg, Timothy E. Wilens, Ty S. Schepis, Vita V. McCabe, Philip T. Veliz

**Affiliations:** 1 Center for the Study of Drugs, Alcohol, Smoking and Health, School of Nursing, University of Michigan, Ann Arbor; 2 Institute for Research on Women and Gender, University of Michigan, Ann Arbor; 3 Institute for Healthcare Policy and Innovation, University of Michigan, Ann Arbor; 4 Injury Prevention Center, University of Michigan, Ann Arbor; 5 Institute for Social Research, University of Michigan, Ann Arbor; 6 Department of Psychology, University of Michigan, Ann Arbor; 7 Department of Psychiatry, School of Medicine, Harvard University, Boston, Massachusetts; 8 Department of Psychology, Texas State University, San Marcos; 9 Department of Psychiatry, Medical School, University of Michigan, Ann Arbor

## Abstract

**Importance:**

Recent information on the prevalence of prescription stimulant therapy for attention-deficit/hyperactivity disorder (ADHD) and nonmedical use of prescription stimulants (NUPS) at the school-level among US secondary school students is limited.

**Objective:**

To investigate the school-level prevalence of and association between stimulant therapy for ADHD and NUPS among US secondary school students.

**Design, Setting, and Participants:**

This cross-sectional study used survey data collected between 2005 and 2020 as part of the Monitoring the Future study (data collected annually via self-administered survey in schools from independent cohorts). Participants were from a nationally representative sample of 3284 US secondary schools. The mean (SD) response rates were 89.5% (1.3%) for 8th-grade students, 87.4% (1.1%) for 10th-grade students, and 81.5% (1.8%) for 12th-grade students. Statistical analysis was performed from July to September 2022.

**Main Outcome and Measure:**

Past-year NUPS.

**Results:**

The 3284 schools contained 231 141 US 8th-, 10th-, and 12th-grade students (111 864 [50.8%, weighted] female; 27 234 [11.8%, weighted] Black, 37 400 [16.2%, weighted] Hispanic, 122 661 [53.1%, weighted] White, 43 846 [19.0%, weighted] other race and ethnicity). Across US secondary schools, the past-year prevalence of NUPS ranged from 0% to more than 25%. The adjusted odds of an individual engaging in past-year NUPS were higher at secondary schools with higher proportions of students who reported stimulant therapy for ADHD, after controlling for other individual-level and school-level covariates. Students attending schools with the highest rates of prescription stimulant therapy for ADHD had approximately 36% increased odds of past-year NUPS compared with students attending schools with no medical use of prescription stimulants (adjusted odds ratio, 1.36; 95% CI, 1.20-1.55). Other significant school-level risk factors included schools in more recent cohorts (2015-2020), schools with higher proportions of parents with higher levels of education, schools located in non-Northeastern regions, schools located in suburban areas, schools with higher proportion of White students, and schools with medium levels of binge drinking.

**Conclusions and Relevance:**

In this cross-sectional study of US secondary schools, the prevalence of past-year NUPS varied widely, highlighting the need for schools to assess their own students rather than relying solely on regional, state, or national results. The study offered new evidence of an association between a greater proportion of the student body that uses stimulant therapy and a greater risk for NUPS in schools. The association between greater school-level stimulant therapy for ADHD and other school-level risk factors suggests valuable targets for monitoring, risk-reduction strategies, and preventive efforts to reduce NUPS.

## Key Points

**Question:**

What is the association between school-level stimulant therapy for attention-deficit/hyperactivity disorder (ADHD) and prescription stimulant nonmedical use?

**Findings:**

In this cross-sectional study of 231 141 students in 3284 secondary schools, school-level past-year prescription stimulant nonmedical use ranged from 0% to more than 25% across US schools. Students attending schools with the highest rates of stimulant therapy for ADHD had 36% increased odds of nonmedical prescription stimulant use compared with students attending schools with the lowest rates.

**Meaning:**

This study’s results suggest potential targets for strategies to reduce school-level prescription stimulant nonmedical use.

## Teaser

This cross-sectional study examines the school-level prevalence of and association between stimulant therapy for attention-deficit/hyperactivity disorder (ADHD) and nonmedical use of prescription stimulants among US secondary school students.

## Introduction

The diagnosis of attention-deficit/hyperactivity disorder (ADHD) and prescribing of stimulant therapy for ADHD have increased substantially in the US over the past 2 decades with recent estimates indicating one in every nine 12th graders report lifetime stimulant therapy for ADHD.^1,2,3,4,5,6^ Although prescription stimulants are efficacious when used appropriately, there is growing concern regarding the acute and long-term adverse health effects (eg, cardiovascular events, depressed mood, overdoses, psychosis, seizures, and stimulant use disorder) associated with nonmedical use of prescription stimulants (NUPS).^7,8,9,10,11^ The leading medication source for NUPS by US adolescents remains same-age peers,^10,12,13,14,15^ and a regional Canadian study^16^ found that the number of secondary school students prescribed stimulants who divert the medication in the classroom is linked to the prevalence of NUPS in that classroom. Thus, the challenge of reducing NUPS while providing necessary stimulant therapy continues to perplex the public health and clinical fields.^17,18,19,20,21^

Despite a call for more attention to school-level contextual influences in adolescent substance use research,^22,23,24,25,26^ little is known about whether school-level stimulant therapy for ADHD is associated with NUPS among US secondary school students. Previous studies have largely neglected school-level factors associated with NUPS among US secondary school students, including school size, school geographical location, school-level racial composition, school-level rates of substance use (eg, binge drinking), and school-level stimulant therapy for ADHD. Research addressing these gaps is needed to identify important sociocontextual influences and intervention targets for NUPS.

To address existing knowledge gaps, this study used data from the Monitoring the Future (MTF) study, a multicohort survey of US nationally representative samples of secondary school students (8th, 10th, and 12th grade) to improve understanding of school-level prevalence and risk factors of NUPS. The main objectives of this study were to examine school-level prevalence rates of NUPS within a large national sample of US secondary school students and determine the school-level characteristics (eg, percentage of students in school prescribed stimulant therapy) and individual-level characteristics (eg, demographic characteristics) associated with NUPS.

## Methods

### Study Design

The MTF study is an annual self-administered survey conducted with nationally representative samples of 8th-, 10th-, and 12th-grade students in US secondary schools.^15^ The 12th-grade sample has been collected since 1975, and the 8th- and 10th-grade samples have been collected since 1991.^15^ Approximately 350 students in each school (for the targeted grade) are selected to participate (either by randomly sampling entire classrooms or by some other unbiased, random method). In schools with smaller numbers of students, the usual procedure is to select all students (for the targeted grade) for participation. The mean (SD) response rates were 89.5% (1.3%) for 8th graders, 87.4% (1.1%) for 10th graders, and 81.5% (1.8%) for 12th graders. The MTF project design, protocol, and sampling methods are described in greater detail elsewhere.^15^

This cross-sectional study used the 2005 to 2020 MTF samples; questions regarding stimulant therapy for ADHD were introduced in 2005. The 2005 to 2020 samples were composed of 231 141 students in 3284 unique public and private schools. The breakdown of schools was 1232 8th-grade schools, 1009 10th-grade schools, and 1043 12th-grade schools. The analytic sample of students consisted of 80 746 8th graders, 78 361 10th graders, and 72 034 12th graders. Schools typically participated for 2 consecutive years in the MTF sample, with 740 participating once, 2462 participating twice, and 82 participating 3 or more times (because they were selected more than once across the 16-year period), yielding 5963 observations at the school-level. There was a mean (SD) of 170.2 (149.7) students per 8th grade school, 249.7 (200.7) students per 10th grade school, and 212.8 (179.3) students per 12th grade school. The questions assessing stimulant therapy for ADHD were asked on only 1 of 4 randomly assigned survey forms for 8th and 10th graders, and 2 of 6 randomly assigned survey forms for 12th graders. Across the years, paper or tablet self-administered surveys have been administered in schools. Informed consent (active or passive, per school policy) was obtained from parents of students younger than 18 years and from students aged 18 years or older, and adolescent assent was obtained. The University of Michigan institutional review board deemed this study exempt because data were recorded at another time by the original investigators and in such a manner that respondents cannot be identified. This study followed the Strengthening the Reporting of Observational Studies in Epidemiology (STROBE) reporting guideline.

### Measures

Current and previous medical use of prescription stimulants was measured at the individual level with an item that assesses medical use of stimulant medications to treat ADHD. Respondents were told stimulant medications are prescribed for people with ADHD who have problems concentrating on one task at a time, or with being too active or too disruptive (hyperactive), or both. Respondents were given a list of generic and brand name stimulant medications (eg, amphetamine, methylphenidate, Ritalin, Adderall, Concerta, Metadate, Dexedrine, Focalin, Vyvanse). Respondents were instructed not to count drugs that are not stimulant-type medications such as Strattera, Wellbutrin, Provigil, Tenex, Intuniv, or Catapres. Respondents were asked if they had ever taken stimulant medications for ADHD under a physician’s or health professional’s supervision. At the individual level, the variable was treated as a 3-category variable: (1) no; (2) yes, in the past, but not now; (3) yes, current use. At the school level, both response options for in the past and current use were combined and aggregated to the school level to reflect the percentage of the study body who used prescription stimulants for ADHD. For sensitivity analysis, we also examined current use at the school level.

Individual-level sociodemographic characteristics included several self-reported measures at the student level, such as sex, race and ethnicity, parental education, and grade point average. Race and ethnicity options were defined by the MTF study team and included Hispanic, non-Hispanic Black, non-Hispanic White, and other (which included American Indian, Asian, and those who selected multiple races and ethnicities). Race and ethnicity were assessed and included based on the well-established race differences in stimulant therapy for ADHD and NUPS. Individual-level substance use included binge drinking, cigarette smoking, and marijuana use.

School-level characteristics were assessed with a set of questions aimed at both the school level and aggregated from measures at the student level consistent with previous work.^25,26,27,28^ The school-level characteristics included school type (public or private), urbanicity (rural, suburban, or urban), US Census region (Northeast, Midwest, South, or West), grade level sampled at school (8th, 10th, or 12th), school size, percentage of the student body that was female, percentage of the student body that had at least 1 parent with a college degree or higher, percentage of the student body that was White, and cohort year (2005 to 2009, 2010 to 2014, 2015 to 2020).

School-level substance use was estimated from student-level variables. It included the percentage of the student body that engaged in past-2-week binge drinking, percentage of the student body that reported past-month cigarette smoking, and the percentage of the student body that reported past-month marijuana use.

NUPS was measured by asking respondents the following item: “On how many occasions (if any) have you taken amphetamines or other prescription stimulant drugs on your own—that is, without a doctor telling you to take them... in your lifetime?...during the last 12 months?...during the last 30 days?” Respondents were given several details about prescription stimulants and informed these medications are prescribed by physicians or health professionals for people who have ADHD and have trouble paying attention, are hyperactive, or both.

### Statistical Analysis

All analyses were design-based using the svyset command in Stata 17.0 (StataCorp), fully accounting for the MTF sampling weights and complex sampling design when estimating parameters for the target MTF individual-level population. The school was treated as the primary sampling unit and was used to correct for the clustering of students within schools. First, we performed descriptive statistics assessing the associations between key school-level characteristics (Table 1) and both past-year NUPS and current and previous medical use of prescription stimulants at the school level. School-level rates of NUPS and medical use were treated as continuous measures for this descriptive analysis. The school-level bivariate analysis did not use the individual-level weights provided by the MTF given that these weights only reflected the 8th-, 10th-, and 12th-grade population in the US and not schools (the MTF does not provide school-level weights). Accordingly, the MTF weights were only used at the individual level.

**Table 1.** Demographics for Individual-Level and School-Level Characteristics

Characteristics	Weighted % (unweighted No.)
Individual-level (n = 231 141)	
Sex	
Female	50.8 (111 864)
Male	49.2 (108 442)
Missing	4.7 (10 835)^a^
Race and ethnicity	
Black (non-Hispanic)	11.8 (27 234)
Hispanic	16.2 (37 400)
White (non-Hispanic)	53.1 (122 661)
Other^b^	19.0 (43 846)
Missing	0.0 (0)^a^
Parental education	
<BA	43.8 (91 637)
BA or higher	51.1 (117 740)
Missing	9.4 (21 764)^a^
Grade point average	
B- or higher	79.4 (175 210)
C+ or lower	20.6 (45 380)
Missing	4.6 (10 551)^a^
Binge drinking (2-week)	
No	86.1 (183 166)
Yes	13.9 (29 487)
Missing	8.0 (18 488)^a^
Cigarette use (30-d)	
No	90.1 (201 762)
Yes	9.9 (22 128)
Missing	3.1 (7251)^a^
Marijuana use (30-d)	
No	85.5 (189 364)
Yes	14.5 (32 209)
Missing	4.1 (9568)^a^
Cohort year	
2005-2009	34.4 (79 599)
2010-2014	32.2 (74 021)
2015-2020	33.5 (77 521)
School level (aggregated)	
No.	5963
% Female^c^	
Low (47 or below)	33.6 (2002)
Medium (48-54)	33.2 (1977)
High (55-100)	33.3 (1983)
% White^c^	
Low (44 or below)	33.4 (1994)
Medium (45-77)	33.8 (2018)
High (78-100)	32.7 (1951)
Parental education (% with a BA or higher)^c^	
Low (45 or below)	33.6 (2005)
Medium (46-66)	33.1 (1974)
High (66-higher)	33.3 (1984)
% With low grades (C+ or lower)^c^	
Low (12 or below)	34.1 (2033)
Medium (13-24)	32.7 (1950)
High (25 or higher)	33.2 (1980)
% Binge drinking^d^	
None (0)	18.3 (1093)
Low (1-9)	26.9 (1604)
Medium (10-19)	27.8 (1656)
High (20 or higher)	27.0 (1610)
% Cigarette smoking^c^	
None (0)	23.7 (1414)
Low (1-7)	25.6 (1525)
Medium (8-15)	25.3 (1509)
High (16 or higher)	25.4 (1515)
% Marijuana use^d^	
None (0)	17.5 (1045)
Low (1-10)	27.5 (1642)
Medium (11-19)	27.5 (1637)
High (20 or higher)	27.5 (1639)
School size^d^	
Small (1-86 students)	33.5 (2000)
Medium (87-252 students)	33.1 (1976)
Large (253 or more)	33.3 (1986)
School level (measured at the school-level) (n = 5963)	
Grade level	
8th	36.7 (2187)
10th	31.0 (1847)
12th	32.3 (1929)
Private status	
Public school	83.1 (4957)
Catholic school	9.4 (558)
Private school	7.5 (448)
Urbanicity of school	
City	31.9 (1903)
Suburban	38.9 (2321)
Rural	29.2 (1739)
US Census region	
Northeast	21.3 (1268)
Midwest	25.5 (1519)
South	33.7 (2012)
West	19.5 (1164)
% Stimulant therapy for ADHD^d^	
None (0)	21.2 (1266)
Low (1-6)	26.9 (1602)
Medium (7-11)	26.1 (1558)
High (12 or higher)	25.8 (1537)
% Nonmedical use of prescription stimulants (past-year)^d^	
None (0)	28.6 (1706)
Low (1-4)	23.8 (1418)
Medium (5-8)	23.9 (1423)
High (9 or higher)	23.7 (1416)

Abbreviation: ADHD, attention-deficit/hyperactivity disorder; BA, bachelor’s degree.

^a^ Percentage missing is based on the total percentage (not valid percentage, which is reported for those who responded to the item of interest). Furthermore, past-year nonmedical use of prescription stimulants had 3.6% item missingness (n = 8240) and stimulant therapy for ADHD had 12.1% item missingness (n = 28 013). There were no missing cases at the school level.

^b^ The other category included American Indian, Asian, and those who selected multiple races and ethnicities.

^c^ Aggregated measures were created to equally divide the sample of schools into tertiles in order to have adequate sample sizes at the school level. Note that the upper ranges (higher-end tertiles) represent the highest prevalence and percentage within the sample of schools.

^d^ Aggregated measures for substance use were created to equally divide the sample of schools into tertiles based on schools that had at least 1 student reporting use of the specific substance.

Second, multivariable logistic regression models were fitted to estimate the association between past-year individual-level NUPS and the individual- and school-level characteristics. Accordingly, 3 sets of models were estimated: one assessed the association between individual-level NUPS and individual-level characteristics, a second assessed the association between individual-level NUPS and school-level characteristics, and a third included both the individual- and school-level characteristics to estimate individual-level NUPS. Finally, an additional set of models was estimated to assess the association between past-year individual-level NUPS and current and previous use of stimulants to treat ADHD at both the individual level and school level. No data were missing for school-level characteristics, and items missing rates for individual-level characteristics ranged from 0.0% for race and ethnicity to 9.4% for parental education; all analyses used listwise deletion. Models using full information maximum likelihood estimation found similar results.

Statistical analysis was performed from July to September 2022. Analyses included 95% CIs to determine statistical significance at a .05 α level or lower.

## Results

As shown in the Figure, there was wide variation across 3284 US secondary schools in the prevalence rates of school-level medical use of prescription stimulants (231 141 US 8th-, 10th-, and 12th-grade students; 111 864 [50.8%, weighted] female; 27 234 [11.8%, weighted] Black, 37 400 [16.2%, weighted] Hispanic, 122 661 [53.1%, weighted] White, 43 846 [19.0%, weighted] other race and ethnicity). The percentage of the student body that reported medical use of prescription stimulants ranged from 1266 schools that had 0% to 116 schools that had 25% or higher. The mean (SD) of medical use of prescription stimulants was 7.9% (7.1%) at the school level, and the mean (SE) at the individual level was 8.0% (0.001%). Additional school-level information: mean (SD) percentage female: 50.9% (0.1%); mean (SD) percentage White: 55.8% (0.3%); mean (SD) percentage of the student body that has at least 1 parent with a college degree or higher: 56.0% (0.2%); mean (SD) percentage with low grades: 19.8% (0.1%); mean (SD) percentage binge drinking: 13.4% (0.1%); mean (SD) percentage cigarette smoking: 10.2% (0.1%); mean (SD) percentage marijuana use: 13.9% (0.1%); mean (SD) percentage nonmedical use of prescription stimulants: 5.7% (0.1%); mean (SD) percentage stimulant therapy for ADHD: 7.9% (0.1%).

**Figure.**
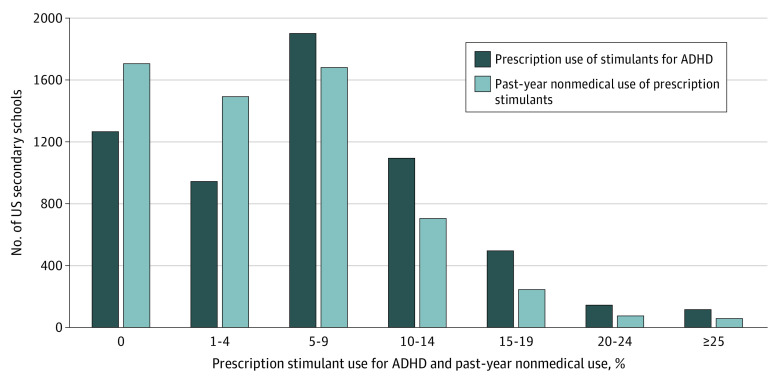
Prevalence of Prescription Stimulant Use and Nonmedical Use Across US Secondary Schools, 2005 to 2020 ADHD indicates attention-deficit/hyperactivity disorder.

As depicted in the Figure, similar to medical use, there was a wide variation across US secondary schools in the past-year prevalence rates of school-level NUPS. Across schools, the percentage of the student body that engaged in past-year NUPS ranged from 1706 schools that had 0% to 58 schools with 25% or higher, including a maximum of 75%. The mean (SD) of past-year NUPS was 5.7% (6.1%) at the school level and mean (SE) of 6.0% (0.001%) at the individual level.

As illustrated in Table 2, the school-level prevalence of past-year NUPS differed significantly as a function of each school-level characteristic. Most notably, schools with a high percentage of the student body using stimulant therapy for ADHD had a higher percentage of the student body engaged in past-year NUPS when compared with schools where none of the students reported medical use of prescription stimulants for ADHD (7.6% [95% CI, 7.3%-8.0%] vs 3.6% [95% CI, 3.2%-3.9%], respectively).

**Table 2.** Mean Percentage of Stimulant Therapy and Nonmedical Use of Prescription Stimulants by Secondary School-Level Characteristics, 2005 to 2020

School-level characteristics	% (95% CI)	
	Past-year nonmedical use of prescription stimulants	Current and past stimulant therapy for ADHD
Private status		
Public school	5.9 (5.7-6.1)	7.8 (7.6-7.9)
Catholic school	4.5 (4.0-5.0)	7.2 (6.5-7.8)
Private school	4.6 (3.9-5.2)	10.2 (9.0-11.2)
Grade level		
8th	3.6 (3.4-3.8)	7.4 (7.1-7.7)
10th	6.5 (6.3-6.8)	7.8 (7.6-8.2)
12th	7.2 (6.8-7.5)	8.5 (8.2-8.9)
Urbanicity of school		
Urban	4.8 (4.6-5.1)	7.2 (6.8-7.4)
Suburban	6.0 (5.8-6.2)	8.5 (8.2-8.8)
Rural	6.1 (5.8-6.4)	8.0 (7.6-8.3)
School size		
Small (1-86 students)	5.0 (4.6-5.3)	7.9 (7.4-8.3)
Medium (87-252)	5.9 (5.6-6.1)	8.2 (8.0-8.5)
High (253 or more)	6.2 (5.9-6.3)	7.6 (7.4-7.8)
US Census region		
Northeast	4.6 (4.4-4.9)	7.0 (6.6-7.4)
Midwest	6.2 (5.8-6.5)	8.0 (7.6-8.3)
South	6.1 (5.8-6.3)	9.3 (9.0-9.6)
West	5.5 (5.1-5.8)	6.4 (6.0-6.8)
% With low grades		
Low (0-12)	5.2 (4.9-5.5)	7.8 (7.5-8.2)
Medium (13-24)	5.8 (5.6-6.1)	8.0 (7.7-8.2)
High (25-100)	6.0 (5.7-6.3)	7.9 (7.6-8.2)
% Female		
Low (0-47)	5.8 (5.5-6.1)	8.5 (8.2-8.8)
Medium (48-54)	5.8 (5.6-6.1)	8.0 (7.7-8.3)
High (55-100)	5.4 (5.1-5.7)	7.3 (6.9-7.6)
Parental education		
Low (0-45)	5.4 (5.2-5.7)	7.3 (7.1-7.6)
Medium (46-66)	6.2 (5.9-6.4)	7.8 (7.5-8.1)
High (67-100)	5.4 (5.2-5.7)	8.6 (8.2-8.9)
% White		
Low (0-44)	4.2 (4.0-4.5)	6.2 (5.9-6.5)
Medium (45-77)	6.1 (5.8-6.3)	8.7 (8.5-9.0)
High (78-100)	6.7 (6.4-7.0)	8.7 (8.4-9.1)
% Binge drinking		
None (0)	2.7 (2.3-3.0)	6.8 (6.3-7.5)
Low (1-9)	3.9 (3.7-4.1)	7.2 (7.0-7.5)
Medium (10-19)	6.2 (5.9-6.4)	8.1 (7.7-8.3)
High (20-100)	8.9 (8.5-9.3)	9.2 (8.8-9.5)
% Cigarette smoking		
None (0)	2.7 (2.4-3.0)	6.7 (6.3-7.2)
Low (1-7)	4.3 (4.1-4.4)	7.1 (6.8-7.3)
Medium (8-15)	6.3 (6.0-6.5)	8.2 (7.9-8.5)
High (16-100)	9.2 (8.8-9.6)	9.5 (9.1-9.9)
% Marijuana use		
None (0)	2.3 (2.1-2.6)	7.1 (6.6-7.7)
Low (1-10)	4.2 (4.1-4.4)	7.3 (7.1-7.6)
Medium (11-19)	6.2 (6.0-6.5)	7.8 (7.5-8.1)
High (20-100)	8.7 (8.3-9.1)	9.1 (8.6-9.4)
% Stimulant therapy for ADHD		
None (0)	3.6 (3.2-3.9)	NA
Low (1-6)	5.1 (4.8-5.3)	NA
Medium (7-11)	6.1 (5.8-6.3)	NA
High (12 or higher)	7.6 (7.3-8.0)	NA
% Nonmedical use of prescription stimulants		
None (0)	NA	6.5 (6.1-6.9)
Low (1-4)	NA	6.9 (6.7-7.2)
Medium (5-8)	NA	8.3 (8.0-8.6)
High (9 or higher)	NA	10.2 (9.8-10.6)

Abbreviations: ADHD, attention-deficit/hyperactivity disorder; NA, not applicable.

As illustrated in Table 3, multivariable regression analyses revealed that all individual-level characteristics were associated with past-year NUPS when adjusting for both individual- and school-level factors (ie, model 3). For instance, adolescents who engaged in past 30-day marijuana use had approximately 4 times greater odds of past-year NUPS when compared with adolescents who did not use marijuana (adjusted odds ratio [aOR], 3.93 [95% CI, 3.69-4.19]). With respect to the school-level characteristics in the fully adjusted model (ie, both individual- and school-level factors; model 3), several characteristics increased the odds of NUPS at the individual level: suburban school location (vs urban), Midwest, South, and West US census region (vs Northeast), higher proportions of 1 or more parents with a college degree, greater percentage of the student body that is White, and higher prevalence of student binge drinking.

**Table 3.** School-Level and Individual-Level Risk Factors of NUPS

Risk factors	Past-year NUPS, aOR (95% CI)		
	Individual-level only, model 1^a^	School-level only, model 2^b^	Full model, model 3^c^
No.	184 157	222 852	184 122
Individual-level			
Sex			
Male	1 [Reference]	NA	1 [Reference]
Female	1.29 (1.23-1.36)	NA	1.31 (1.24-1.38)
Race and ethnicity			
White	1 [Reference]	NA	1 [Reference]
Black	0.52 (0.46-0.58)	NA	0.56 (0.50-0.64)
Hispanic	0.74 (0.68-0.80)	NA	.83 (0.75-0.90)
Other	0.95 (0.88-1.02)	NA	1.01 (0.94-1.09)
Highest parental education			
<BA	1 [Reference]	NA	1 [Reference]
BA or higher	0.97 (0.92-1.02)	NA	0.94 (0.89-0.99)
Grade point average			
B- or higher	1 [Reference]	NA	1 [Reference]
C+ or lower	1.34 (1.26-1.43)	NA	1.33 (1.25-1.42)
Binge drinking			
No	1 [Reference]	NA	1 [Reference]
Yes	2.79 (2.61-2.97)	NA	2.76 (2.58-2.95)
Cigarette smoking			
No	1 [Reference]	NA	1 [Reference]
Yes	3.19 (2.98-3.41)	NA	3.12 (2.91-3.35)
Marijuana use			
No	1 [Reference]	NA	1 [Reference]
Yes	3.90 (3.66-4.15)	NA	3.93 (3.69-4.19)
Cohort year (individual/school)			
2005-2009	1 [Reference]	1 [Reference]	1 [Reference]
2010-2014	1.05 (.98-1.13)	1.07 (1.01-1.13)	1.08 (1.01-1.16)
2015-2020	1.16 (1.08-1.25)	1.17 (1.10-1.25)	1.24 (1.14-1.35)
School-level			
Private status			
Public school	NA	1 [Reference]	1 [Reference]
Catholic school	NA	1.04 (0.91-1.20)	1.09 (0.93-1.30)
Private school	NA	1.01 (0.87-1.19)	1.04 (0.87-1.24)
Grade level			
8th	NA	1 [Reference]	1 [Reference]
10th	NA	1.03 (0.96-1.10)	1.01 (0.93-1.10)
12th	NA	0.88 (0.82-0.96)	0.82 (0.74-.91)
Urbanicity of school			
Urban	NA	1 [Reference]	1 [Reference]
Suburban	NA	1.05 (1.00-1.11)	1.08 (1.01-1.16)
Rural	NA	1.01 (0.94-1.09)	1.00 (.91-1.11)
School size			
Small (1-86 students)	NA	1 [Reference]	1 [Reference]
Medium (87-252 students)	NA	0.91 (0.88-1.00)	0.99 (0.88-1.09)
Large (253 or more)	NA	0.95 (0.86-1.04)	1.06 (0.94-1.20)
US Census region			
Northeast	NA	1 [Reference]	1 [Reference]
Midwest	NA	1.27 (1.17-1.37)	1.36 (1.23-1.50)
South	NA	1.32 (1.23-1.42)	1.52 (1.38-1.67)
West	NA	1.36 (1.25-1.48)	1.40 (1.26-1.56)
% With low grades			
Low (0-12)	NA	1 [Reference]	1 [Reference]
Medium (13-24)	NA	1.02 (.96-1.08)	0.95 (0.88-1.03)
High (25-100)	NA	0.99 (0.93-1.06)	0.90 (0.82-0.98)
% Female			
Low (0-47)	NA	1 [Reference]	1 [Reference]
Medium (48-54)	NA	1.04 (0.99-1.10)	1.04 (0.98-1.11)
High (55-100)	NA	1.05 (1.00-1.11)	1.00 (0.94-1.08)
Parental education			
Low (0-45)	NA	1 [Reference]	1 [Reference]
Medium (46-66)	NA	1.07 (.99-1.16)	1.17 (1.09-1.26)
High (67-100)	NA	1.08 (.98-1.20)	1.12 (1.02-1.23)
% White			
Low (0- 44)	NA	1 [Reference]	1 [Reference]
Medium (45-77)	NA	1.14 (1.07-1.21)	1.03 (0.95-1.11)
High (78-100)	NA	1.12 (1.04-1.20)	1.14 (1.03-1.26)
% Binge drinking			
None (0)	NA	1 [Reference]	1 [Reference]
Low (1-9)	NA	1.19 (1.05-1.35)	1.10 (0.95-1.28)
Medium (10-19)	NA	1.48 (1.30-1.69)	1.22 (1.03-1.44)
High (20-100)	NA	1.73 (1.51-1.99)	1.17 (0.99-1.40)
% Cigarette smoking			
None (0)	NA	1 [Reference]	1 [Reference]
Low (1-7)	NA	1.12 (1.01-1.24)	0.91 (0.80-1.02)
Medium (8-15)	NA	1.39 (1.25-1.55)	0.97 (0.85-1.11)
High (16-100)	NA	1.79 (1.59-2.02)	1.02 (0.88-1.18)
% Marijuana use			
None (0)	NA	1 [Reference]	1 [Reference]
Low (1-10)	NA	1.49 (1.30-1.72)	1.16 (0.99-1.37)
Medium (11-19)	NA	1.82 (1.57-2.10)	1.14 (0.96-1.36)
High (20-100)	NA	2.24 (1.92-2.61)	1.14 (0.96-1.38)
% Stimulant therapy for ADHD			
None (0)	NA	1 [Reference]	1 [Reference]
Low (1-6)	NA	1.18 (1.08-1.30)	1.29 (1.14-1.45)
Medium (7-11%)	NA	1.31 (1.19-1.44)	1.39 (1.23-1.57)
High (12 or higher)	NA	1.54 (1.40-1.70)	1.64 (1.45-1.86)

Abbreviations: ADHD, attention-deficit/hyperactivity disorder; aOR, adjusted odds ratio; BA, bachelor’s degree; NA, not applicable (variable not included in the model); NUPS, nonmedical use of prescription stimulants.

^a^ Model 1 only includes individual-level risk factors when assessing individual-level past-year NUPS.

^b^ Model 2 only includes school-level risk factors when assessing individual-level past-year NUPS.

^c^ Model 3 includes both individual-level and school-level risk factors when assessing individual-level past-year NUPS.

Moreover, in Table 3 (models 2 and 3), 12th graders had lower odds of past-year NUPS when compared with 8th graders (the opposite was true in the bivariate analyses in Table 2). Finally, of central importance and similar to what was found in the bivariate analyses, adolescents that attended schools with a higher percentage of the student body who indicated using stimulant therapy for ADHD had higher odds of past-year NUPS. For instance, the odds of an adolescent who attended a school with a high percentage of the student body who indicated using stimulant therapy for ADHD had approximately one-and-a-half times greater odds of NUPS when compared with adolescents who attended a school where no students used stimulant therapy for ADHD (aOR, 1.64 [95% CI, 1.45-1.86]). Supplemental analyses found this association was significant for past-month NUPS (see eTable 1 in Supplement 1) and after controlling for the percentage of students engaging in NUPS at the school-level (see eTable 2 in Supplement 1). Table 4 shows that both the individual-level and school-level factors assessing stimulant therapy for ADHD were associated with past-year NUPS. For instance, model 2 in Table 4 shows that adolescents who indicated either current (aOR, 2.34 [95% CI, 2.09-2.61]) or past (aOR, 2.44 [95% CI, 2.22-2.67]) stimulant therapy for ADHD had approximately two-and-one-half times greater odds of past-year NUPS when compared with their peers who never used stimulants to treat ADHD. Moreover, adolescents who attended schools with a higher percentage of the student body who used stimulant therapy for ADHD had higher odds of NUPS when also controlling for medical use at the individual level. For instance, the adjusted odds of an adolescent who attended a school with a high percentage of the student body who reported stimulant therapy for ADHD had approximately 36% greater odds of NUPS when compared with adolescents who attended a school where no students used stimulant therapy for ADHD (aOR, 1.36 [95% CI, 1.20-1.55]). Stratified analyses showed that these findings remained robust for students who did not currently or had not previously used stimulant therapy for ADHD (aOR, 1.33 [95% CI, 1.16-1.52]) (eTable 3 in Supplement 1). Finally, sensitivity analyses found similar results for both current and previous use as well as for continuous school-level factors (see eTables 4, 5, 6, 7, and 8 in Supplement 1).

**Table 4.** School-Level and Individual-Level Risk Factors of Past-Year NUPS, Including Stimulant Therapy History

Risk factors	Past-year NUPS, aOR (95% CI)	
	Individual-level, model 1^a^ (n = 169 823)	Individual-level, model 2^b^ (n = 169 788)
Individual level		
Stimulant therapy for ADHD history		
Never	1 [Reference]	1 [Reference]
Previous use	2.60 (2.37-2.85)	2.44 (2.22-2.67)
Current use	2.51 (2.25-2.81)	2.34 (2.09-2.61)
School level		
% Of students reporting stimulant therapy for ADHD		
None	NA	1 [Reference]
Low	NA	1.23 (1.08-1.39)
Medium	NA	1.27 (1.12-1.45)
High	NA	1.36 (1.20-1.55)

Abbreviations: ADHD, attention-deficit/hyperactivity disorder; aOR, adjusted odds ratio; NA, not applicable (variable not included in the model); NUPS, nonmedical use of prescription stimulants.

^a^ Model 1 controls for sex, race and ethnicity, parental education level, grade point average, past 2-week binge drinking, past 30-day cigarette smoking, past 30-day marijuana use and cohort year. Model 1 shows the results without controlling for school-level risk factors.

^b^ Model 2 controls for sex, race and ethnicity, parental education level, grade point average, past 2-week binge drinking, past 30-day cigarette smoking, past 30-day marijuana use, cohort year, school type, grade level, urbanicity of school, school size, US Census region, percentage of student body with low grades, percentage of the study body that is female, percentage of the student body with at least 1 parent with a college degree or higher, percentage of the student body that is White, percentage of the student body that has engaged in binge drinking during the past 2 weeks, percentage of the student body that has engaged in cigarette smoking during the past 30 days, and the percentage of the student body that has engaged in marijuana use during the past 30 days. This analysis also includes individual-level medical use of stimulant therapy for ADHD history to estimate individual-level past-year NUPS (the analyses in Table 3 did not include this individual-level risk factor of stimulant therapy for ADHD). Model 2 includes both individual-level and school-level risk factors to assess individual-level past-year NUPS.

## Discussion

To our knowledge, this is the first national study to examine school-level prevalence and risk factors associated with NUPS among US secondary school students. Past-year NUPS prevalence across US secondary schools varied considerably, with a range of 0% to 25% or higher of the student body engaging in NUPS. The present study extends previous research that found similar variation in past-year NUPS prevalence across US colleges (0%-25%).^29^ The wide variation found between US secondary schools in the present study serves as notice that individual schools are strongly encouraged to assess their own student body to guide their prevention efforts. Of central importance, based on controlled analyses, the present study found that school-level NUPS was varied significantly with school-level stimulant therapy for ADHD, after adjusting for individual- and school-level risk factors including other school-level substance use.

This is, to our knowledge, the first study to provide detailed national estimates of the association between school-level stimulant therapy for ADHD as an environmental risk factor and school-level NUPS. School-level stimulant therapy had a significant association with individual- and school-level NUPS, indicating a valuable potential target for monitoring, risk-reduction strategies, and prevention. This is especially important given that peers are a leading source for NUPS among adolescents.^10,14^ The study is consistent with previous evidence from a regional Canadian study that found significant associations between the number of students receiving prescription stimulants who share medications in a secondary school classroom and NUPS prevalence in each classroom.^16^

There is growing evidence that the majority of NUPS among older adolescents is primarily motivated by a desire to enhance academics and/or cognition and most often involves obtaining stimulants from friends at the same school.^7,12,16,30,31,32,33^ NUPS is associated with an increased risk of substance use disorder, neuropsychological dysfunction, polysubstance use, depressed mood, and lower graduation rates.^11,34,35,36,37,38^ The findings from this study indicate a need for more research to examine whether closely monitoring medical availability of prescription stimulants and reducing stimulant diversion among same-age peers could have a significant influence on reducing NUPS. However, exerting medication management control and monitoring measures at both the individual- and school-level becomes more challenging after high school when adolescents often become more responsible for their own medication management. Adolescents who are prescribed stimulant therapy for ADHD should be educated and prepared that nearly a quarter (24%) will be approached to divert their stimulant medications by their peers before the completion of high school (and more than half [54%] during college).^37,38^

We sought to better understand what was linked with NUPS with a multilevel approach to examine the association between individual- and school-level risk factors and NUPS during adolescence. In these controlled analyses, it was found that the adjusted odds of past-year NUPS were higher at schools in more recent cohorts (2015 to 2020), schools located in non-Northeastern regions, schools located in suburban areas, schools that had proportionally more White students, and schools that had higher proportions of parents with more education. The higher odds of NUPS in more recent cohorts could be driven by recent increases in stimulant therapy. Clearly, the school context, including the attitudes and behaviors of fellow students regarding the stimulant therapy for ADHD, NUPS, and other substances in general, sets the stage for schoolwide NUPS. Secondary schools vary in terms of how much they are “academic-focused” (ie, high academics, low substance use, and high social integration) and “party-focused” (ie, low academics, high substance use, and low social integration).^39^ The present study found schools that were high on NUPS were also higher on other substance use; nonetheless, when school-level other substance use was controlled for, the association between stimulant therapy for ADHD and NUPS remained strong. We also controlled for numerous individual- and school-level sociodemographics, and together, the association between stimulant therapy and NUPS remained significant.

### Strengths and Limitations

This study had strengths and limitations. The main strength of the present study is the nationally representative sample of 8th-, 10th-, and 12th-grade students attending US public and private schools with large enough samples to derive school-level estimates of stimulant therapy for ADHD and NUPS. It is also worth noting that large national studies, such as MTF, also carry limitations, such as not including students who were home-schooled, dropped out, or were absent on the day of data collection which can all affect estimates. Although the MTF study measures have been reported to be reliable and valid, there is some evidence that adolescents may misclassify or underreport sensitive substance use behaviors.^15,40,41,42^

## Conclusion

This cross-sectional study found that there is a wide variation in the prevalence of NUPS between US secondary schools (0% to more than 25%), highlighting the need for individual schools to assess their own student body rather than relying solely on regional, state, or national results. To our knowledge, this is the first national study to identify school-level risk factors associated with NUPS among US secondary school students. These findings suggest that school-level stimulant therapy for ADHD and other school-level risk factors were significantly associated with NUPS and should be accounted for in risk-reduction strategies and prevention efforts.

## Notes

Supplement 1. **eTable 1.** School-Level and Individual-Level Correlates of Past-Month Nonmedical Use of Prescription Stimulants **eTable 2.** School-Level and Individual-Level Correlates of Nonmedical Use of Prescription Stimulants (NUPS) Controlling for School-Level NUPS **eTable 3.** School-Level and Individual-Level Correlates of Nonmedical Use of Prescription Stimulants Among Those Without Stimulant Therapy **eTable 4.** School-Level and Individual-Level Correlates of Nonmedical Use of Prescription Stimulants With Continuous School-Level Correlates (With Additional Characteristics) **eTable 5.** School-Level and Individual-Level Correlates of Nonmedical Use of Prescription Stimulants With Continuous School-Level Correlates **eTable 6.** Bivariate Correlations Between Stimulant Therapy for ADHD and Nonmedical Use of Prescription Stimulants by Secondary School-Level Characteristics, 2005–2020 **eTable 7.** Demographics for School-Level Characteristics **eTable 8.** Demographics for Individual-Level Characteristics

Supplement 2.
Data Sharing Statement
