# Supplementary material for: Prescription Stimulant Medical and Nonmedical Use Among US Secondary School Students, 2005 to 2020
Source: JAMA Netw Open. 2023 Apr 18;6(4):e238707. doi: 10.1001/jamanetworkopen.2023.8707 (PMC10114020; doi:10.1001/jamanetworkopen.2023.8707)
Supplement: Supplement 1. — eTable 1. School-Level and Individual-Level Correlates of Past-Month Nonmedical Use of Prescription Stimulants eTable 2. School-Level and Individual-Level Correlates of Nonmedical Use of Prescription Stimulants (NUPS) Controlling for School-Level NUPS eTable 3. School-Level and Individual-Level Correlates of Nonmedical Use of Prescription Stimulants Among Those Without Stimulant Therapy eTable 4. School-Level and Individual-Level Correlates of Nonmedical Use of Prescription Stimulants With Continuous School-Level Correlates (With Additional Characteristics) eTable 5. School-Level and Individual-Level Correlates of Nonmedical Use of Prescription Stimulants With Continuous School-Level Correlates eTable 6. Bivariate Correlations Between Stimulant Therapy for ADHD and Nonmedical Use of Prescription Stimulants by Secondary School-Level Characteristics, 2005–2020 eTable 7. Demographics for School-Level Characteristics eTable 8. Demographics for Individual-Level Characteristics [file jamanetwopen-e238707-s001.pdf]

Supplemental Online Content

McCabe SE, Schulenberg JE, Wilens TE, Schepis TS, McCabe VV, Veliz PT. Prescription stimulant medical and nonmedical use among US secondary school students, 2005 to 2020. *JAMA Netw Open.* 2023;6(4):e238707. doi:10.1001/jamanetworkopen.2023.8707

eTable 1. School-Level and Individual-Level Correlates of Past-Month Nonmedical Use of Prescription Stimulants

eTable 2. School-Level and Individual-Level Correlates of Nonmedical Use of Prescription Stimulants (NUPS) Controlling for School-Level NUPS

eTable 3. School-Level and Individual-Level Correlates of Nonmedical Use of Prescription Stimulants Among Those Without Stimulant Therapy

eTable 4. School-Level and Individual-Level Correlates of Nonmedical Use of Prescription Stimulants With Continuous School-Level Correlates (With Additional Characteristics)

eTable 5. School-Level and Individual-Level Correlates of Nonmedical Use of Prescription Stimulants With Continuous School-Level Correlates

eTable 6. Bivariate Correlations Between Stimulant Therapy for ADHD and Nonmedical Use of Prescription Stimulants by Secondary School-Level Characteristics, 2005–2020

eTable 7. Demographics for School-Level Characteristics

eTable 8. Demographics for Individual-Level Characteristics

This supplemental material has been provided by authors to give readers additional information about their work.

© 2023 McCabe SE et al. *JAMA Network Open.*

eTable 1. School-level and individual-level correlates of past-month nonmedical use of prescription stimulants

Correlates	Past-month NUPS (n = 184,120) Model 1[a] AOR (95% CI)	Past-month NUPS (n = 222,788) Model 2[b] AOR (95% CI)	Past-month NUPS (n = 184,085) Model 3[c] AOR (95% CI)
Individual-level			
Sex			
Male	REF	---	REF
Female	1.30 (1.21, 1.40)***	---	1.32 (1.22, 1.42)***
Race/ethnicity			
White	REF	---	REF
Black	.741 (.639, .858)***	---	.774 (.660, .906)***
Hispanic	.801 (.716, .896)***	---	.856 (.755, .970)*
Other	.996 (.899, 1.10)	---	1.03 (.935, 1.15)
Highest parental education			
Less than a BA	REF	---	REF
BA or higher	.990 (.918, 1.06)	---	.966 (.893, 1.04)
Grade point average			
B- or higher	REF	---	REF
C+ or lower	1.31 (1.20, 1.42)***	---	1.27 (1.16, 1.39)***
Binge drinking			
No	REF	---	REF
Yes	3.13 (2.87, 3.42)***	---	3.17 (2.89, 3.47)***
Cigarette smoking			
No	REF	---	REF
Yes	3.00 (2.76, 3.28)***	---	2.92 (2.66, 3.20)***
Marijuana use			
No	REF	---	REF
Yes	4.17 (3.83, 4.54)***	---	4.41 (4.03, 4.82)***
Cohort year (individual/school)			
2005–2009	REF	REF	REF
2010–2014	1.02 (.933, 1.12)	1.04 (.970, 1.13)	1.06 (.973, 1.17)
2015–2020	1.14 (1.03, 1.25)**	1.17 (1.07, 1.28)***	1.24 (1.11, 1.38)***
School-level			
Private status			
Public school	---	REF	REF
Catholic school	---	.903 (.751, 1.08)	.952 (.767, 1.18)
Private school	---	.993 (.796, 1.23)	1.08 (.848, 1.39)
Grade level			
8th	---	REF	REF
10th	---	.976 (.890, 1.07)	.968 (.863, 1.08)
12th	---	.783 (.702, .873)***	.731 (.637, .839)***
Urbanicity of school			
Urban	---	REF	REF
Suburban	---	1.02 (.946, 1.10)	1.02 (.932, 1.11)
Rural	---	.999 (.901, 1.10)	1.00 (.886, 1.13)
School size			
Small (1 to 86 students)	---	REF	REF
Medium (87 to 252 students)	---	.973 (.862, 1.09)	1.11 (.966, 1.29)
Large (253 or more)	---	.942 (.824, 1.07)	1.12 (.963, 1.32)
US Census region			
Northeast	---	REF	REF

© 2023 McCabe SE et al. *JAMA Network Open.*

Correlates	Past-month NUPS (n = 184,120) Model 1[a] AOR (95% CI)	Past-month NUPS (n = 222,788) Model 2[b] AOR (95% CI)	Past-month NUPS (n = 184,085) Model 3[c] AOR (95% CI)
Midwest	---	1.26 (1.14, 1.40)***	1.34 (1.19, 1.52)***
South	---	1.32 (1.20, 1.46)***	1.51 (1.35, 1.70)***
West	---	1.30 (1.16, 1.46)***	1.37 (1.19, 1.57)***
Percent with low grades			
Low (0% to 12%)	---	REF	REF
Medium (13% to 24%)	---	.988 (.905, 1.07)	.920 (.827, 1.02)
High (25% to 100%)	---	.967 (.872, 1.07)	.904 (.797, 1.02)
Percent female			
Low (0% to 47%)	---	REF	REF
Medium (48% to 54%)	---	1.05 (.973, 1.13)	1.03 (.946, 1.13)
High (55% to 100%)	---	1.01 (.937, 1.10)	.953 (.862, 1.05)
Parental education			
Low (0% to 45%)	---	REF	REF
Medium (46% to 66%)	---	1.08 (.996, 1.17)	1.08 (.977, 1.19)
High (67% to 100%)	---	1.10 (.998, 1.22)	1.09 (.967, 1.23)
Percent White			
Low (0% to 44%)	---	REF	REF
Medium (45% to 77%)	---	1.05 (.961, 1.15)	.987 (.881, 1.10)
High (78% to 100%)	---	1.18 (1.06, 1.32)***	1.08 (.950, 1.24)
Percent binge drinking			
None (0%)	---	REF	REF
Low (1% to 9%)	---	1.12 (.931, 1.35)	.999 (.793, 1.25)
Medium (10% to 19%)	---	1.42 (1.17, 1.73)***	1.10 (.863, 1.41)
High (20% to 100%)	---	1.70 (1.38, 2.10)***	1.08 (.834, 1.41)
Percent cigarette smoking			
None (0%)	---	REF	REF
Low (1% to 7%)	---	1.21 (1.04, 1.41)**	.988 (.826, 1.18)
Medium (8% to 15%)	---	1.54 (1.32, 1.80)***	1.05 (.871, 1.27)
High (16% to 100%)	---	2.01 (1.70, 2.38)***	1.12 (.911, 1.38)
Percent marijuana use			
None (0%)	---	REF	REF
Low (1% to 10%)	---	1.50 (1.21, 1.85)***	1.08 (.846, 1.38)
Medium (11% to 19%)	---	1.87 (1.51, 2.32)***	1.00 (.780, 1.30)
High (20% to 100%)	---	2.25 (1.80, 2.81)***	.943 (.718, 1.23)
Percent stimulant therapy for ADHD			
None (0%)	---	REF	REF
Low (1% to 6%)	---	1.22 (1.07, 1.40)**	1.29 (1.10, 1.52)***
Medium (7% to 11%)	---	1.28 (1.12, 1.46)***	1.31 (1.11, 1.54)***
High (12% or higher)	---	1.57 (1.37, 1.80)***	1.59 (1.35, 1.87)***

Abbreviations: NUPS, nonmedical use of prescription stimulants; AOR, adjusted odds ratio; CI, confidence interval; BA, bachelor's degree; ADHD, attention-deficit/hyperactivity disorder.

Notes:

"REF" refers to the reference category. "---" refers to variable not included in the model. Sample sizes vary because of missing data.

[a]Model 1 only includes individual-level predictors/correlates when assessing individual-level past 30-day NUPS.

[b]Model 2 only includes school-level predictors/correlates when assessing individual-level past 30-day NUPS.

[c]Model 3 includes both individual-level and school-level predictors/correlates when assessing individual-level past 30-day NUPS.

*$P < .05$, **$P < .01$, ***$P < .001$. *Indicates a significant difference at the .05 alpha level or lower, **Indicates a significant difference at the .01 alpha level or lower, and ***Indicates a significant difference at the .001 alpha level or lower.

© 2023 McCabe SE et al. *JAMA Network Open.*

eTable 2. School-level and individual-level correlates of nonmedical use of prescription stimulants (NUPS) controlling for school-level NUPS

Correlates	Past-year NUPS (n = 184,157) Model 1[a] AOR (95% CI)	Past-year NUPS (n = 222,852) Model 2[b] AOR (95% CI)	Past-year NUPS (n = 184,122) Model 3[c] AOR (95% CI)
Individual-level			
Sex			
Male	REF	---	REF
Female	1.29 (1.23, 1.36)***	---	1.32 (1.25, 1.39)***
Race/ethnicity			
White	REF	---	REF
Black	.519 (.461, .584)***	---	.597 (531, .671)***
Hispanic	.735 (.677, .799)***	---	.834 (.765, .911)***
Other	.946 (.879, 1.02)	---	1.02 (.948, 1.10)
Highest parental education			
Less than a BA	REF	---	REF
BA or higher	.972 (.921, 1.02)	---	.932 (.882, .985)*
Grade point average			
B- or higher	REF	---	REF
C+ or lower	1.34 (1.26, 1.43)***	---	1.35 (1.26, 1.44)***
Binge drinking			
No	REF	---	REF
Yes	2.79 (2.61, 2.97)***	---	2.89 (2.70, 3.10)***
Cigarette smoking			
No	REF	---	REF
Yes	3.19 (2.98, 3.41)***	---	3.30 (3.07, 3.54)***
Marijuana use			
No	REF	---	REF
Yes	3.90 (3.66, 4.15)***	---	4.13 (3.87, 4.41)***
Cohort year (individual/school)			
2005–2009	REF	REF	REF
2010–2014	1.05 (.984, 1.13)	1.05 (1.01, 1.08)**	1.07 (1.01, 1.12)**
2015–2020	1.16 (1.08, 1.25)***	1.08 (1.04, 1.13)***	1.14 (1.08, 1.22)***
School-level			
Private status			
Public school	---	REF	REF
Catholic school	---	.998 (.925, 1.08)	1.04 (.943, 1.15)
Private school	---	.974 (.886, 1.07)	.970 (.858, 1.09)
Grade level			
8th	---	REF	REF
10th	---	.984 (.949, 1.02)	.971 (.917, 1.02)
12th	---	.951 (.907, .997)*	.897 (.833, .967)***
Urbanicity of school			
Urban	---	REF	REF
Suburban	---	.997 (.964, 1.03)	1.02 (.967, 1.06)
Rural	---	.970 (.925, 1.01)	.972 (.908, 1.04)
School size			
Small (1 to 86 students)	---	REF	REF
Medium (87 to 252 students)	---	.995 (.939, 1.05)	1.06 (.990, 1.15)
Large (253 or more)	---	.959 (.899, 1.02)	1.07 (.990, 1.17)
US Census region			
Northeast	---	REF	REF

© 2023 McCabe SE et al. *JAMA Network Open.*

Correlates	Past-year NUPS (n = 184,157) Model 1[a] AOR (95% CI)	Past-year NUPS (n = 222,852) Model 2[b] AOR (95% CI)	Past-year NUPS (n = 184,122) Model 3[c] AOR (95% CI)
Midwest	---	1.07 (1.03, 1.12)***	1.13 (1.05, 1.21)***
South	---	1.07 (1.03, 1.12)***	1.20 (1.13, 1.28)***
West	---	1.10 (1.05, 1.15)***	1.11 (1.03, 1.19)**
Percent with low grades			
Low (0% to 12%)	---	REF	REF
Medium (13% to 24%)	---	1.01 (.939, 1.05)	.943 (.895, .994)*
High (25% to 100%)	---	1.01 (.970, 1.05)	.915 (.857, .977)**
Percent female			
Low (0% to 47%)	---	REF	REF
Medium (48% to 54%)	---	1.00 (.975, 1.03)	1.00 (.953, 1.05)
High (55% to 100%)	---	1.00 (.967, 1.03)	.952 (.904, 1.00)
Parental education			
Low (0% to 45%)	---	REF	REF
Medium (46% to 66%)	---	1.03 (.999, 1.06)	1.05 (1.00, 1.11)*
High (67% to 100%)	---	1.04 (.996, 1.08)	1.04 (.978, 1.12)
Percent White			
Low (0% to 44%)	---	REF	REF
Medium (45% to 77%)	---	.987 (.949, 1.02)	.889 (.838, .943)***
High (78% to 100%)	---	1.04 (1.00, 1.09)*	.904 (.841, .972)**
Percent binge drinking			
None (0%)	---	REF	REF
Low (1% to 9%)	---	1.15 (1.07, 1.24)***	1.07 (.957, 1.19)
Medium (10% to 19%)	---	1.14 (1.05, 1.23)***	.917 (.814, 1.03)
High (20% to 100%)	---	1.21 (1.11, 1.31)***	.776 (.682, .882)***
Percent cigarette smoking			
None (0%)	---	REF	REF
Low (1% to 7%)	---	1.10 (1.07, 1.24)***	.888 (.821, .961)**
Medium (8% to 15%)	---	1.12 (1.05, 1.23)***	.756 (.693, .826)***
High (16% to 100%)	---	1.22 (1.14, 1.31)***	.654 (.590, .725)***
Percent marijuana use			
None (0%)	---	REF	REF
Low (1% to 10%)	---	1.25 (1.15, 1.35)***	.973 (.872, 1.08)
Medium (11% to 19%)	---	1.29 (1.19, 1.41)***	.790 (.703, .888)***
High (20% to 100%)	---	1.34 (1.22, 1.46)***	.639 (.562, .726)***
Percent past-year NUPS			
Low (0% to 4%)	---	REF	REF
Medium (5% to 8%)	---	3.34 (3.20, 3.48)***	3.60 (3.40, 3.81)***
High (9% or higher)	---	6.64 (6.34, 6.95)***	8.19 (7.69, 8.73)***
Percent stimulant therapy for ADHD			
None (0%)	---	REF	REF
Low (1% to 6%)	---	1.07 (1.02, 1.13)**	1.16 (1.07, 1.27)***
Medium (7% to 11%)	---	1.08 (1.03, 1.14)**	1.13 (1.04, 1.24)**
High (12% or higher)	---	1.17 (1.10, 1.23)***	1.21 (1.11, 1.33)***

Abbreviations: AOR, adjusted odds ratio; CI, confidence interval; BA, bachelor's degree; ADHD, attention-deficit/hyperactivity disorder.

Notes:

"REF" refers to the reference category. "---" refers to variable not included in the model. Sample sizes vary because of missing data.

[a]Model 1 only includes individual-level predictors/correlates when assessing individual-level past-year NUPS.

[b]Model 2 only includes school-level predictors/correlates when assessing individual-level past-year NUPS.

[c]Model 3 includes both individual-level and school-level predictors/correlates when assessing individual-level past-year NUPS.

*$P < .05$, **$P < .01$, ***$P < .001$. *Indicates a significant difference at the .05 alpha level or lower, **Indicates a significant difference at the .01 alpha level or lower, and ***Indicates a significant difference at the .001 alpha level or lower.

© 2023 McCabe SE et al. *JAMA Network Open.*

eTable 3. School-level and individual-level correlates of nonmedical use of prescription stimulants among those without stimulant therapy

Correlates	Past-year stimulant misuse (n = 156,953)	Past-year stimulant misuse (n = 184,086)	Past-year stimulant misuse (n = 156,919)
	Model 1[a]	Model 2[b]	Model 3[c]
	AOR (95% CI)	AOR (95% CI)	AOR (95% CI)
Individual-level			
Sex			
Male	REF	---	REF
Female	1.30 (1.23, 1.38)***	---	1.32 (1.24, 1.40)***
Race/ethnicity			
White	REF	---	REF
Black	.518 (.451, .595)***	---	.550 (.475, .636)***
Hispanic	.747 (.680, .822)***	---	.809 (.728, .899)***
Other	.916 (.839, 1.00)	---	.965 (.881, 1.05)
Highest parental education			
Less than a BA	REF	---	REF
BA or higher	.954 (.898, 1.01)	---	.934 (.877, .995)*
Grade point average			
B- or higher	REF	---	REF
C+ or lower	1.29 (1.20, 1.39)***	---	1.28 (1.18, 1.38)***
Binge drinking			
No	REF	---	REF
Yes	2.80 (2.60, 3.02)***	---	2.77 (2.57, 2.99)***
Cigarette smoking			
No	REF	---	REF
Yes	3.23 (2.98, 3.50)***	---	3.16 (2.91, 3.43)***
Marijuana use			
No	REF	---	REF
Yes	3.99 (3.71, 4.29)***	---	4.00 (3.71, 4.31)***
Cohort year (individual/school)			
2005–2009	REF	REF	REF
2010–2014	1.03 (.955, 1.12)	1.06 (.994, 1.13)	1.06 (.982, 1.15)
2015–2020	1.14 (1.05, 1.24)***	1.15 (1.06, 1.24)***	1.25 (1.14, 1.38)***
School-level			
Private status			
Public school	---	REF	REF
Catholic school	---	1.03 (.887, 1.20)	1.05 (.879, 1.26)
Private school	---	1.00 (.816, 1.22)	1.01 (.807, 1.28)
Grade level			
8th	---	REF	REF
10th	---	.997 (.919, 1.08)	.979 (.885, 1.08)
12th	---	.872 (.793, .959)**	.820 (.728, .924)***
Urbanicity of school			
Urban	---	REF	REF
Suburban	---	1.04 (.974, 1.11)	1.07 (.988, 1.16)
Rural	---	1.01 (.923, 1.11)	1.01 (.903, 1.13)
School size			
Small (1 to 86 students)	---	REF	REF
Medium (87 to 252 students)	---	.953 (.855, 1.60)	1.03 (.913, 1.17)
Large (253 or more)	---	1.00 (.888, 1.13)	1.12 (.976, 1.29)
US Census region			
Northeast	---	REF	REF

© 2023 McCabe SE et al. *JAMA Network Open.*

Correlates	Past-year stimulant misuse (n = 156,953) Model 1[a] AOR (95% CI)	Past-year stimulant misuse (n = 184,086) Model 2[b] AOR (95% CI)	Past-year stimulant misuse (n = 156,919) Model 3[c] AOR (95% CI)
Midwest	---	1.34 (1.22, 1.46)***	1.42 (1.28, 1.59)***
South	---	1.41 (1.30, 1.54)***	1.60 (1.43, 1.78)***
West	---	1.44 (1.30, 1.59)***	1.48 (1.31, 1.68)***
Percent with low grades			
Low (0% to 12%)	---	REF	REF
Medium (13% to 24%)	---	1.01 (.945, 1.09)	.964 (.884, 1.05)
High (25% to 100%)	---	.985 (.905, 1.07)	.906 (.817, 1.00)
Percent female			
Low (0% to 47%)	---	REF	REF
Medium (48% to 54%)	---	1.06 (1.00, 1.13)*	1.05 (.978, 1.13)
High (55% to 100%)	---	1.03 (.967, 1.10)	.986 (.909, 1.07)
Parental education			
Low (0% to 45%)	---	REF	REF
Medium (46% to 66%)	---	1.13 (1.06, 1.22)***	1.17 (1.07, 1.28)***
High (67% to 100%)	---	1.11 (1.02, 1.21)*	1.10 (.991, 1.22)
Percent White			
Low (0% to 44%)	---	REF	REF
Medium (45% to 77%)	---	1.16 (1.08, 1.26)***	1.03 (.939, 1.14)
High (78% to 100%)	---	1.33 (1.22, 1.46)***	1.14 (1.02, 1.28)*
Percent binge drinking			
None (0%)	---	REF	REF
Low (1% to 9%)	---	1.16 (1.00, 1.35)*	1.11 (.929, 1.34)
Medium (10% to 19%)	---	1.53 (1.31, 1.79)***	1.29 (1.07, 1.57)**
High (20% to 100%)	---	1.78 (1.51, 2.10)***	1.22 (.994, 1.50)
Percent cigarette smoking			
None (0%)	---	REF	REF
Low (1% to 7%)	---	1.07 (.952, 1.21)	.879 (.761, 1.01)
Medium (8% to 15%)	---	1.35 (1.18, 1.54)***	.983 (.840, 1.15)
High (16% to 100%)	---	1.75 (1.51, 2.30)***	1.03 (.872, 1.23)
Percent marijuana use			
None (0%)	---	REF	REF
Low (1% to 10%)	---	1.57 (1.33, 1.85)***	1.22 (1.02, 1.47)*
Medium (11% to 19%)	---	1.95 (1.64, 2.31)***	1.21 (1.00, 1.46)*
High (20% to 100%)	---	2.43 (2.03, 2.91)***	1.21 (.992, 1.48)
Percent stimulant therapy for ADHD			
None (0%)	---	REF	REF
Low (1% to 6%)	---	1.08 (.974, 1.20)	1.18 (1.04, 1.35)**
Medium (7% to 11%)	---	1.13 (1.02, 1.26)*	1.24 (1.09, 1.41)***
High (12% or higher)	---	1.17 (1.05, 1.31)*	1.33 (1.16, 1.52)***

Abbreviations: ADHD, attention-deficit/hyperactivity disorder; AOR, adjusted odds ratio; CI, confidence interval; BA, bachelor's degree; NUPS, nonmedical use of prescription stimulants.

Notes:

"REF" refers to the reference category. "---" refers to variable not included in the model. Sample sizes vary because of missing data.

[a]Model 1 only includes individual-level predictors/correlates when assessing individual-level past-year NUPS.

[b]Model 2 only includes school-level predictors/correlates when assessing individual-level past-year NUPS.

[c]Model 3 includes both individual-level and school-level predictors/correlates when assessing individual-level past-year NUPS.

*$P < .05$, **$P < .01$, ***$P < .001$. *Indicates a significant difference at the .05 alpha level or lower, **Indicates a significant difference at the .01 alpha level or lower, and ***Indicates a significant difference at the .001 alpha level or lower.

© 2023 McCabe SE et al. *JAMA Network Open.*

eTable 4. School-level and individual-level correlates of nonmedical use of prescription stimulants with continuous school-level correlates (with additional characteristics)

Correlates	Past-year NUPS (n = 184,157)	Past-year NUPS (n = 222,846)	Past-year NUPS (n = 184,121)
	Model 1[a]	Model 2[b]	Model 3[c]
	AOR (95% CI)	AOR (95% CI)	AOR (95% CI)
Individual-level			
Sex			
Male	REF	---	REF
Female	1.29 (1.23, 1.36)***	---	1.31 (1.24, 1.38)***
Race/ethnicity			
White	REF	---	REF
Black	.519 (.461, .584)***	---	.571 (504, .646)***
Hispanic	.735 (.677, .799)***	---	.844 (.770, .925)***
Other	.946 (.879, 1.02)	---	1.01 (.940, 1.09)
Highest parental education			
Less than a BA	REF	---	REF
BA or higher	.972 (.921, 1.02)	---	.933 (.882, .986)*
Grade point average			
B- or higher	REF	---	REF
C+ or lower	1.34 (1.26, 1.43)***	---	1.34 (1.25, 1.43)***
Binge drinking			
No	REF	---	REF
Yes	2.79 (2.61, 2.97)***	---	2.81 (2.63, 3.00)***
Cigarette smoking			
No	REF	---	REF
Yes	3.19 (2.98, 3.41)***	---	3.14 (2.93, 3.37)***
Marijuana use			
No	REF	---	REF
Yes	3.90 (3.66, 4.15)***	---	3.92 (3.68, 4.18)***
Cohort year (individual/school)			
2005–2009	REF	REF	REF
2010–2014	1.05 (.984, 1.13)	1.06 (1.00, 1.13)*	1.06 (.992, 1.14)
2015–2020	1.16 (1.08, 1.25)***	1.11 (1.04, 1.19)**	1.18 (1.08, 1.28)***
School-level			
Private status			
Public school	---	REF	REF
Catholic school	---	.960 (.835, 1.10)	1.00 (.852, 1.19)
Private school	---	.947 (.726, .989)*	.875 (.731, 1.04)
Grade level			
8th	---	REF	REF
10th	---	1.14 (1.06, 1.22)***	1.08 (.993, 1.17)
12th	---	.946 (.870, 1.02)	.872 (.786, .967)**
Urbanicity of school			
Urban	---	REF	REF
Suburban	---	1.07 (1.01, 1.13)*	1.08 (1.01, 1.16)*
Rural	---	.990 (.913, 1.07)	.981 (.887, 1.08)
School size (continuous)	---	1.00 (1.00, 1.00)***	1.00 (1.00, 1.00)*
US Census region			
Northeast	---	REF	REF
Midwest	---	1.30 (1.20, 1.40)***	1.36 (1.23, 1.50)***

© 2023 McCabe SE et al. *JAMA Network Open.*

Correlates	Past-year NUPS (n = 184,157) Model 1[a] AOR (95% CI)	Past-year NUPS (n = 222,846) Model 2[b] AOR (95% CI)	Past-year NUPS (n = 184,121) Model 3[c] AOR (95% CI)
South	---	1.42 (1.31, 1.53)***	1.53 (1.39, 1.69)***
West	---	1.38 (1.27, 1.51)***	1.40 (1.25, 1.56)***
Percent with low grades (continuous)	---	1.04 (.828, 1.31)	.701 (.517, .950)*
Percent female (continuous)	---	1.32 (1.06, 1.64)*	.958 (.730, 1.25)
Parental education (continuous)	---	1.13 (.958, 1.33)	1.19 (.962, 1.48)
Percent White (continuous)	---	1.70 (1.51, 1.90)***	1.27 (1.09, 1.49)**
Percent binge drinking (continuous)	---	2.94 (2.18, 3.98)***	.981 (.666, 1.44)
Percent cigarette use (continuous)	---	5.95 (4.14, 8.54)***	1.12 (.711, 1.76)
Percent marijuana use (continuous)	---	6.61 (4.98, 8.78)***	1.17 (.802, 1.71)
Percent stimulant therapy for ADHD (continuous)	---	7.37 (4.90, 11.1)***	10.8 (16.65, 17.5)***

Abbreviations: NUPS, nonmedical use of prescription stimulants; AOR, adjusted odds ratio; CI, confidence interval; BA, bachelor's degree; ADHD, attention-deficit/hyperactivity disorder.

Notes:

"REF" refers to the reference category. "---" refers to variable not included in the model. Sample sizes vary because of missing data.

[a]Model 1 only includes individual-level predictors/correlates when assessing individual-level past-year NUPS.

[b]Model 2 only includes school-level predictors/correlates when assessing individual-level past-year NUPS.

[c]Model 3 includes both individual-level and school-level predictors/correlates when assessing individual-level past-year NUPS.

*$P < .05$, **$P < .01$, ***$P < .001$. *Indicates a significant difference at the .05 alpha level or lower, **Indicates a significant difference at the .01 alpha level or lower, and ***Indicates a significant difference at the .001 alpha level or lower.

© 2023 McCabe SE et al. *JAMA Network Open.*

eTable 5. School-level and individual-level correlates of nonmedical use of prescription stimulants with continuous school-level correlates

Correlates	Past-year NUPS (n = 169,823)	Past-Year NUPS (n = 169,788)
	Model 1[a]	Model 2[b]
	AOR (95% CI)	AOR (95% CI)
Individual-level		
Stimulant therapy for ADHD history		
Never	REF	REF
Previous use	2.60 (2.37, 2.85)***	3.56 (3.31, 3.82)***
Current use	2.51 (2.25, 2.81)***	3.18 (2.92, 3.46)***
School-level		
Percent of students reporting stimulant therapy for ADHD (continuous)	---	1.66 (1.05, 2.63)*

Abbreviations: NUPS, nonmedical use of prescription stimulants; AOR, adjusted odds ratio; CI, confidence interval; ADHD, attention-deficit/hyperactivity disorder.

Notes:

“REF” refers to the reference category. “---” refers to variable not included in the model.

[a]Model 1 controls for sex, race, highest parental education level, grade point average, past two-week binge drinking, past 30-day cigarette use, past 30-day marijuana use and cohort year. Model 1 shows the results without controlling for school-level predictors/correlates.

[b]Model 2 and 3 controls for sex, race, highest parental education level, grade point average, past two-week binge drinking, past 30-day cigarette use, past 30-day marijuana use, cohort year, school type, grade level, urbanicity of school, school size, US Census region, percent of student body with low grades, percent of the study body that is female, percent of the student body with at least one parent with a college degree or higher, percent of the student body that is White, percent of the student body that has engaged in binge drinking during the past two weeks, percent of the student body that has engaged in cigarette use during the past 30 days, and the percent of the student body that has engaged in marijuana use during the past 30 days. This analysis includes individual-level medical use of stimulant therapy for ADHD history to predict individual-level past-year NUPS (the analyses in Table 3 did not include this individual-level predictor/correlate of stimulant therapy for ADHD). Model 2 and 3 includes both individual-level and school-level predictors/correlates to assess individual-level past-year NUPS.

*$P < .05$, **$P < .01$, ***$P < .001$. *Indicates a significant difference at the .05 alpha level or lower, **Indicates a significant difference at the .01 alpha level or lower, and ***Indicates a significant difference at the .001 alpha level or lower.

© 2023 McCabe SE et al. *JAMA Network Open.*

eTable 6. Bivariate correlations between prescription stimulant therapy for ADHD and nonmedical use of prescription stimulants by secondary school-level characteristics, 2005–2020

School-level characteristics	Current and past stimulant therapy for ADHD b (β)	*P* value[a]	Past-year NUPS b (β)	*P* value[a]
Private status				
Public school	REF	REF	REF	REF
Catholic school	-.006 (-.026)	.044	-.014 (-.067)	< .001
Private school	.023 (.086)	< .001	-.013 (-.057)	< .001
Grade level				
8th	REF	REF	REF	REF
10th	.004 (.030)	< .001	.029 (.224)	< .001
12th	.011 (.077)	< .001	.035 (.274)	< .001
Urbanicity of school				
Urban	REF	REF	REF	REF
Suburban	.013 (.093)	<.001	.012 (.097)	<.001
Rural	.008 (.053)	<.001	.013 (.098)	<.001
School size	-.001 (-.030)	.018	.001 (.063)	<.001
US Census region				
Northeast	REF	REF	REF	REF
Midwest	.009 (.058)	< .001	.015 (.109)	< .001
South	.022 (.152)	< .001	.014 (.111)	< .001
West	-.006 (-.034)	.028	.008 (.055)	< .001
Percent with low grades	.003 (.007)	.559	.026 (.060)	.559
Percent female	-.045 (-.089)	< .001	-.020 (-.047)	< .001
Parental education	.027 (.086)	< .001	-.001 (-.001)	.917
Percent White	.037 (.167)	< .001	.035 (.185)	< .001
Percent binge drinking	.080 (.140)	< .001	.195 (.399)	< .001
Percent cigarette smoking	.108 (.163)	< .001	.239 (.424)	< .001
Percent marijuana use	.074 (.126)	< .001	.204 (.413)	< .001
Percent stimulant therapy for ADHD	NA	NA	.202 (.237)	< .001
Percent nonmedical use of prescription stimulants	.278 (.237)	< .001	NA	NA

Abbreviations: ADHD, attention-deficit/hyperactivity disorder; NUPS, nonmedical use of prescription stimulants; b, unstandardized regression coefficient; β, standardized regression coefficient; REF, reference; NA, not applicable.
[a]Ordinary least squares (OLS) regression models were used to determine the standardized regression coefficient.

© 2023 McCabe SE et al. *JAMA Network Open.*

eTable 7. Demographics for school-level characteristics

**School-level (measured at the school-level; continuous) (n = 5963)**	**Mean (SD)**
Percent female	50.9% (13.8%)
Percent White	55.9% (31.8%)
Parental education (% with a BA or higher)	56.0% (22.3%)
Percent with low grades (C+ or lower)	19.8% (14.0%)
Percent binge drinking	13.4% (12.4%)
Percent cigarette smoking	10.2% (10.7%)
Percent marijuana use	13.9% (12.2%)
Percent stimulant therapy for ADHD	7.9% (7.1%)
Percent nonmedical use of prescription stimulants (past-year)	5.7% (6.0%)
School size	208.6 (179.3)
**School-level (measured at the school-level; categorical) (n = 5963)**	**% (n)**
Grade level	
8th	36.7 (2187)
10th	31.0 (1847)
12th	32.3 (1929)
Private status	
Public school	83.1 (4957)
Catholic school	9.4 (558)
Private school	7.5 (448)
Urbanicity of school	
City	31.9 (1903)
Suburban	38.9 (2321)
Rural	29.2 (1739)
US Census region	
Northeast	21.3 (1268)
Midwest	25.5 (1519)
South	33.7 (2012)
West	36.7 (2187)

Abbreviations: SD, standard deviation; BA, bachelor’s degree; ADHD, attention-deficit/hyperactivity disorder.
Notes:
% = weighted percent; n = unweighted sample size.

© 2023 McCabe SE et al. *JAMA Network Open.*

eTable 8. Demographics for individual-level and school-level characteristics

**Individual-level (n = 231,141)**	**Current and past stimulant therapy for ADHD**		**Past-year NUPS**	
	**% (n)**	***P* value**[a]	**% (n)**	***P* value**[a]
Sex				
Female	6.5 (6709)	REF	6.1 (6755)	REF
Male	9.3 (8929)	< .001	5.7 (6171)	.004
Race/ethnicity				
White	9.2 (10578)	REF	6.9 (8592)	REF
Non-White	6.3 (5689)	< .001	4.7 (4777)	< .001
Parental education				
Less than a BA	7.5 (6217)	REF	6.7 (6081)	REF
BA or higher	8.3 (9018)	< .001	5.6 (6592)	< .001
Grade point average				
B- or higher	6.9 (11136)	REF	5.0 (8728)	REF
C+ or lower	12.2 (4820)	< .001	9.5 (4278)	< .001
Binge drinking (2-week)				
No	6.7 (11127)	REF	3.3 (6188)	REF
Yes	13.3 (3516)	< .001	21.3 (6095)	< .001
Cigarette use (30-day)				
No	6.9 (12512)	REF	3.7 (7548)	REF
Yes	16.9 (3390)	< .001	26.1 (5677)	< .001
Marijuana use (30-day)				
No	6.9 (11721)	REF	3.1 (5929)	REF
Yes	13.6 (3910)	< .001	22.7 (7159)	< .001
Current and past stimulant therapy for ADHD				
No	NA	NA	5.1 (9417)	REF
Yes	NA	NA	16.7 (2645)	< .001
Past-year NUPS				
No	7.0 (13203)	REF	NA	NA
Yes	21.9 (2645)	< .001	NA	NA
Cohort year				
2005–2009	8.3 (5783)	REF	6.4 (4936)	REF
2010–2014	8.0 (5427)	.282	6.2 (4460)	.309
2015–2020	7.6 (5057)	.002	5.2 (3973)	< .001

Abbreviations: ADHD, attention-deficit/hyperactivity disorder; NUPS, nonmedical use of prescription stimulants; REF, reference; BA, bachelor's degree; NA, not applicable.
Notes:
% = weighted percent; n = unweighted sample size.
[a]Binary logistic regression models were used to determine statistically significant differences between groups.

© 2023 McCabe SE et al. *JAMA Network Open.*
