# Supplementary material for: Prescription Stimulant Medical and Nonmedical Use Among US Secondary School Students, 2005 to 2020
Source: JAMA Netw Open. 2023 Apr 18;6(4):e238707. doi: 10.1001/jamanetworkopen.2023.8707 (PMC10114020; doi:10.1001/jamanetworkopen.2023.8707)
Supplement: Supplement 2. — Data Sharing Statement [file jamanetwopen-e238707-s002.pdf]

Data Sharing Statement

McCabe. Prescription Stimulant Medical and Nonmedical Use Among US Secondary School Students, 2005 to 2020. *JAMA Netw Open*. Published April 18, 2023.
doi:10.1001/jamanetworkopen.2023.8707

Data
Data available: No

Additional Information
Explanation for why data not available: This study contains the results of secondary analysis of the USA Monitoring the Future (MTF) surveys. The authors followed university and MTF protocol regarding access to and analysis of the data for this study. Data is not available without written consent from MTF and interested researchers can apply for MTF panel data access through the US National Addiction & HIV Data Archive Program (NAHDAP) at the University of Michigan. More information may be found here:
https://www.icpsr.umich.edu/web/NAHDAP/studies/37072
